# Impact of cytopenias and early versus late treatment with ruxolitinib in patients with steroid-refractory acute or chronic graft-versus-host disease

**DOI:** 10.1038/s41409-024-02445-6

**Published:** 2024-11-06

**Authors:** Zahra Mahmoudjafari, Valkal Bhatt, John Galvin, Zhenyi Xue, Robert Zeiser, Franco Locatelli, Gérard Socié, Mohamad Mohty

**Affiliations:** 1 University of Kansas Cancer Center, Westwood, KS USA; 2 Incyte Corporation, Wilmington, DE USA; 3 University Medical Center Freiburg, Freiburg, Germany; 4 IRCCS Ospedale Pediatrico Bambino Gesù, Rome, Italy; 5 Catholic University of the Sacred Heart, Rome, Italy; 6 Hôpital Saint-Louis Hematology Transplantation & University Paris Cité, Paris, France; 7 Hôpital Saint-Antoine Hospital and Sorbonne University, Paris, France

**Keywords:** Graft-versus-host disease, Allotransplantation

## Abstract

REACH2 and REACH3 were randomized, multicenter, open-label phase 3 studies comparing the selective Janus kinase (JAK)1/JAK2 inhibitor ruxolitinib versus investigators’ choice of best available therapy (BAT) in steroid-refractory (SR) acute (REACH2) or chronic (REACH3) graft-versus-host disease (aGVHD/cGVHD). Moderate-severe aGVHD/cGVHD can progress rapidly; thus, key clinical considerations driving management of patients with SR-aGVHD/SR-cGVHD are prompt treatment initiation and concomitant cytopenias. These post hoc analyses of REACH2/REACH3 describe the impact of timing of treatment initiation after SR-aGVHD/SR-cGVHD diagnosis and development of concomitant cytopenias on treatment outcomes. Ruxolitinib initiation within 3 days from SR-aGVHD diagnosis yielded an extended duration of response and higher Day 28 complete response rates compared with initiation ≥7 days after SR-aGVHD diagnosis (median 178 vs 167 days and 36.6% vs 25.0%, respectively). For patients with SR-cGVHD, Week 24 overall response was not impacted by time to treatment (54.5% vs 42.6% for <14 vs >28 days). Clinically relevant cytopenias were manageable, allowing for maintenance of dose intensity (median 20 mg/d), and did not impact the favorable efficacy outcomes from ruxolitinib treatment. This analysis highlights the practical importance of considering earlier ruxolitinib initiation after SR diagnosis in GVHD and the benefits of ruxolitinib treatment compared with BAT even for patients with cytopenias.

## Introduction

Graft-versus-host disease (GVHD) is a leading cause of morbidity and mortality in patients who undergo allogeneic hematopoietic stem cell transplantation (HSCT) [1–3]. Despite prophylactic immunosuppressive therapies, GVHD develops in ≈50% of allogeneic HSCT recipients. Acute GVHD (aGVHD) mainly affects skin, gastrointestinal (GI) tract, and liver, whereas chronic GVHD (cGVHD) commonly presents in the mouth, genitalia, liver, lungs, skin, GI tract, joints, and muscles [4, 5].

Corticosteroids are the current standard of care for initial GVHD therapy [4, 6–9]. Approximately 30–60% of patients with GVHD become refractory to treatment with corticosteroids [1, 10, 11]. Among patients with steroid-refractory (SR)-aGVHD, the 6-month survival rate is approximately 50% [8], whereas overall survival for patients with SR-cGVHD is approximately 72% at 3 years [12, 13]. Systemic corticosteroids have known severe treatment-related toxicities that limit long-term use [14].

For GVHD after failure of at least one line of systemic therapy, 3 therapies have been approved by the US Food and Drug Administration (FDA) since 2017: ibrutinib and belumosudil are approved for treatment of cGVHD only, and the selective Janus kinase 1/2 (JAK1/JAK2) inhibitor ruxolitinib is approved for treatment of both aGVHD and cGVHD [15]. Per the prescribing information, the GVHD indications for ruxolitinib are SR-aGVHD in adult and pediatric patients ≥12 years and cGVHD after failure of 1–2 lines of systemic therapy in adult and pediatric patients ≥12 years, and NCCN Clinical Practice Guidelines in Oncology (NCCN Guidelines^®^) list ruxolitinib as a category 1 systemic agent for both SR-aGVHD and SR-cGVHD [16].

Ruxolitinib has been studied in randomized, open-label phase 3 studies in both aGVHD (REACH2) and cGVHD (REACH3). REACH2 compared efficacy and safety of ruxolitinib with investigators’ choice of best available therapy (BAT) in 309 patients with SR-aGVHD [14]. Overall response rate (ORR) was significantly higher at Day 28 for ruxolitinib compared with BAT (62% vs 39%, respectively; *P* < 0.001) [14]. Median failure-free survival was also significantly longer in patients treated with ruxolitinib compared with those treated with BAT (5.0 vs 1.0 month, respectively) [14]. REACH3 compared efficacy and safety of ruxolitinib with investigators’ choice of BAT in 329 patients with SR-cGVHD [1]. ORR and best overall response (BOR) up to Week 24 were significantly higher for ruxolitinib versus BAT (ORR, 49.7% vs 25.6%, *P* < 0.001; BOR, 76.4% vs 60.4%, *P* = 0.001) [1]. Patients who received ruxolitinib had significantly longer median failure-free survival versus those who received BAT (REACH3, >18.6 vs 5.7 months; *P* < 0.001; REACH3 3-year follow-up, 38.4 vs 5.7 months); this finding is consistent with a significantly higher response (24.2% vs 11.0%; *P* = 0.001) on the modified Lee Symptom Scale at 24 weeks, a predictor of longer overall survival and nonrelapse mortality [1, 17].

The REACH2/3 safety profiles were consistent with the well-established long-term safety profile of ruxolitinib [18]. Severe grade ≥3 adverse event rates were similar for the 2 treatments (REACH2: ruxolitinib [78%], BAT [78%]; REACH3: ruxolitinib [57%], BAT [57.6%]). Ruxolitinib treatment has been associated with cytopenias, and the most common grade ≥3 adverse events in both REACH trials were thrombocytopenia (REACH2, ruxolitinib [27%], BAT [15%]; REACH3, ruxolitinib [15.2%], BAT [10.1%]) and anemia (REACH2, ruxolitinib [22%], BAT [19%]; REACH3, ruxolitinib [12.7%], BAT [7.6%]) [1, 14]. Anemia was also the most common grade ≥3 event in the 3-year safety analysis of REACH3 (ruxolitinib [17.6%], BAT [9.5%]), and thrombocytopenia occurred at rates ≥5% higher for ruxolitinib than BAT [19].

Because hematologic events are a manifestation of GVHD pathogenesis, ruxolitinib treatment for aGVHD/cGVHD should be optimized for patients with cytopenias. Significant symptomatology and rapid organ progression may be associated with aGVHD/cGVHD, requiring prompt recognition of SR disease and initiation of second-line therapy. Timing of treatment initiation might affect GVHD treatment outcomes and is an important factor in GVHD management [20]. The post hoc analyses of the REACH2 and REACH3 trials presented here evaluated the impact of timing of treatment initiation after SR diagnosis and effect of concomitant cytopenias on treatment response in patients with SR-aGVHD and SR-cGVHD.

## Methods

### REACH2 and REACH3 clinical studies

REACH2 (NCT02913261) and REACH3 (NCT03112603) phase 3 clinical studies compared efficacy and safety of ruxolitinib with investigator-selected BAT in patients with GVHD [1, 14]. Patients from REACH2 had grade II–IV SR-aGVHD. Patients from REACH3 had moderate or severe SR or steroid-dependent cGVHD, collectively referred to as patients with SR-cGVHD. Both studies were multicenter, randomized, open-label, and enrolled patients ≥12 years old who had received an allogeneic HSCT.

Patients were randomized in a 1:1 ratio to 10 mg ruxolitinib twice daily or investigator-selected BAT. All patients continued to receive corticosteroids with/without calcineurin inhibitors. The REACH trials were designed/conducted in accordance with the International Council for Harmonisation Guideline for Good Clinical Practice, Declaration of Helsinki, and local regulatory requirements. Study protocols were approved by institutional review boards at each site. Patients or their guardians provided written informed consent.

### Patient subgroups for treatment initiation timing analysis

To analyze the effect of treatment initiation times on outcomes, patients were divided into subgroups based on time from SR-GVHD diagnosis to treatment initiation. SR-aGVHD subgroups were early (0–3 days), late (4–6 days), and very late ( ≥ 7 days). SR-cGVHD subgroups were early ( ≤ 14 days), late (15–28 days), and very late ( > 28 days). For some analyses, late and very-late subgroups were combined. Subgroups were selected to be congruent with and applicable to current clinical practice for patients with aGVHD and cGVHD [21, 22].

### Patient subgroups for cytopenia analysis

Cytopenia analysis was done only with patients with SR-aGVHD, based on subgroups defined by thresholds for platelet (PLT), hemoglobin (Hb), white blood cell count (WBC), or absolute neutrophil count (ANC) measured between baseline and Week 4. Low blood count thresholds were defined as: <30 × 10^9^ cells/L (PLT); <8 g/dL (Hb); <5 × 10^9^ cells/L (WBC); <1 × 10^9^ cells/L (ANC). Thresholds were selected to be clinically relevant for guiding treatment decision-making and supportive care interventions for patients with GVHD [23].

### Endpoints and assessments

Post hoc analyses evaluated (1) ORR, defined as the percentage of patients who had complete or partial responses (CR/PR, per REACH2/3 [1, 14]) at Day 28 (with SR-aGVHD) or Week 24 (with SR-cGVHD); (2) BOR, defined as the percentage of patients with CR or PR at any time; and (3) duration of response (DOR), defined for SR-aGVHD as time from first response until aGVHD progression or new systemic therapy and for SR-cGVHD as the time from first documented CR/PR among patients with a response at Week 24 until cGVHD progression, death, or additional systemic cGVHD therapies. For DOR, competing events for patients with SR-aGVHD were onset of cGVHD or death without progression of aGVHD; no competing events were defined for SR-cGVHD DOR.

In REACH2, patients with SR-aGVHD who received ≥1 dose of trial treatment were evaluated for the impact of cytopenias on treatment responses and laboratory values. Durable response was defined as the percentage of patients in each SR-aGVHD treatment group who maintained a Day 28 response at Day 56.

### Statistical analysis

For comparative analyses between ruxolitinib treatment versus BAT or between ruxolitinib-only treatment initiation groups, odds ratios and 95% Wald CIs for ORR (or BOR) were calculated using the stratified Cochran-Mantel-Haenszel test. For REACH2, DOR was plotted by cumulative incidence curve, and median and quartiles were estimated using the Kaplan-Meier method. For REACH3, DOR was analyzed using the Kaplan-Meier method.

## Results

### Baseline characteristics of patients with SR-aGVHD

Demographics and clinical characteristics of patients with SR-aGVHD are shown by subgroups in Table 1. Of 309 patients, 154 (49.8%) patients were allocated to the ruxolitinib arm and categorized as receiving treatment early (*n* = 112 [72.7%]), late-and-very-late, (*n* = 42 [27.3%; late, *n* = 18, very late, *n* = 24]), or very late (*n* = 24 [15.6%]). The remaining 155 (50.2%) patients were randomized to BAT and categorized as receiving treatment early (*n* = 115 [74.2%]), late-and-very-late (*n* = 40 [25.8%; late, *n* = 18, very late, *n* = 22]), or very late (*n* = 22 [14.2%]). Mean time from diagnosis of initial aGVHD grade ≥II to randomization for treatment (ruxolitinib or BAT) of SR-aGVHD was 22.4, 37.5, and 42.0 days for the early, late-and-very-late, and very-late subgroups, respectively. Median ages were similar across subgroups, and most patients were men (Table 1).

**Table 1:** Baseline characteristics of patients with SR-aGVHD.

Variable	Early (0–3 days) *N* = 227			Late and Very Late (≥4 days) *N* = 82			Very Late (≥7 days) *N* = 46		
	RUX (*n* = 112)	BAT (*n* = 115)	All (*N* = 227)	RUX (*n* = 42)	BAT (*n* = 40)	All (*N* = 82)	RUX (*n* = 24)	BAT (*n* = 22)	All (*N* = 46)
Age, median (range), y	52.5 (15–73)	54.0 (13–71)	54.0 (13–73)	52.5 (12–69)	55.0 (13–69)	53.5 (12–69)	48.5 (18–62)	52.0 (13–69)	51.0 (13–69)
Men, *n* (%)	62 (55.4)	68 (59.1)	130 (57.3)	30 (71.4)	23 (57.5)	53 (64.6)	18 (75.0)	9 (40.9)	27 (58.7)
aGVHD grade,^a^ *n* (%)									
0	1 (0.9)	0	1 (0.4)	3 (7.1)	1 (2.5)	4 (4.9)	3 (12.5)	1 (4.5)	4 (8.7)
I	2 (1.8)	0	2 (0.9)	0	0	0	0	0	0
II	42 (37.5)	37 (32.2)	79 (34.8)	8 (19.0)	17 (42.5)	25 (30.5)	3 (12.5)	11 (50.0)	14 (30.4)
III	45 (40.2)	51 (44.3)	96 (42.3)	23 (54.8)	17 (42.5)	40 (48.8)	13 (54.2)	9 (40.9)	22 (47.8)
IV	22 (19.6)	27 (23.5)	49 (21.6)	8 (19.0)	5 (12.5)	13 (15.9)	5 (20.8)	1 (4.5)	6 (13.0)
Time from prior aGVHD grade ≥II diagnosis, mean (SD), days^a,b^	25.1 (45.0)	19.8 (32.8)	22.4 (39.3)	41.6 (37.6)	33.1 (24.9)	37.5 (32.1)	46.0 (33.4)	37.6 (26.7)	42.0 (30.3)
Organ involvement^a^ *n* (%)									
Skin	74 (66.1)	55 (47.8)	129 (56.8)	19 (45.2)	19 (47.5)	38 (46.3)	11 (45.8)	9 (40.9)	20 (43.5)
Liver	21 (18.8)	21 (18.3)	42 (18.5)	15 (35.7)	5 (12.5)	20 (24.4)	11 (45.8)	3 (13.6)	14 (30.4)
Upper GI	21 (18.8)	30 (26.1)	51 (22.5)	7 (16.7)	7 (17.5)	14 (17.1)	4 (16.7)	4 (18.2)	8 (17.4)
Lower GI	67 (59.8)	85 (73.9)	152 (67.0)	29 (69.0)	30 (75.0)	59 (72.0)	15 (62.5)	15 (68.2)	30 (65.2)
Missing	1 (0.9)	0	1 (0.4)	3 (7.1)	1 (2.5)	4 (4.9)	3 (12.5)	1 (4.5)	4 (8.7)

*aGVHD* acute graft-versus-host disease, *BAT* best available therapy, *GI* gastrointestinal, *RUX* ruxolitinib, *SR-aGVHD* steroid-refractory acute graft-versus-host disease, *SD* standard deviation, *y* years.

Race and ethnicity data are reported in REACH2 study [14].

^a^At time of randomization/treatment initiation.

^b^Time between diagnosis of initial aGVHD (not steroid refractory) and the randomization for treatment of SR-aGVHD.

At randomization, most patients had grade II or III SR-aGVHD, with more patients having grade III than grade II SR-aGVHD across all treatment initiation subgroups (34.8%, 30.5%, and 30.4% had grade II SR-aGVHD in the early, late-and-very-late, and very-late subgroups, respectively, vs 42.3%, 48.8%, and 47.8% with grade III SR-aGVHD). Skin and lower GI tract organs were most frequently involved; (56.8%, 46.3%, and 43.5% had skin involvement in the early, late-and-very-late, and very-late subgroups, respectively; 67.0%, 72.0%, and 65.2% had lower GI tract involvement).

Patients with SR-aGVHD who received ≥1 dose of trial treatment (*N* = 302) were included in the safety analysis, which evaluated the impact of cytopenias (ruxolitinib, *n* = 152 [50.3%]; BAT, *n* = 150 [49.7%]).

### Baseline characteristics of patients with SR-cGVHD

Demographics and clinical characteristics of the 329 patients with SR-cGVHD are shown in Table 2. Of these patients, 165 (50.2%) were randomized to ruxolitinib and categorized as receiving treatment early (*n* = 88 [53.3%]), late-and-very-late (*n* = 77 [46.7%; late, *n* = 30, very late, *n* = 47]), and very late (*n* = 47 [28.5%]). The remaining 164 (49.8%) patients were randomized to BAT and categorized as receiving treatment early (*n* = 85 [51.8%]), late-and-very-late (*n* = 79 [48.2%; late, *n* = 33, very late, *n* = 46]), and very late (*n* = 46 [28.0%]). Mean time from diagnosis of cGVHD to randomization of ruxolitinib or BAT was 175.7, 290.1, and 323.2 days for early, late-and-very-late, and very-late subgroups, respectively. Median ages for the early, late-and-very-late, and very-late subgroups were all 49.0 years. Most patients were men (59.5%, 62.8%, and 64.5% for the early, late-and-very-late, and very-late subgroups, respectively).

**Table 2:** Baseline characteristics of patients with SR-cGVHD.

Variable	Early (≤14 days) *N* = 173			Late and Very Late (>14 days) *N* = 156			Very Late (>28 days) *N* = 93		
	RUX *n* = 88	BAT *n* = 85	All *N* = 173	RUX *n* = 77	BAT *n* = 79	All *N* = 156	RUX *n* = 47	BAT *n* = 46	All *N* = 93
Age, median (range), y	48.0 (13–72)	51.0 (13–76)	49.0 (13–76)	50.0 (15–73)	47.0 (12–72)	49.0 (12–73)	49.0 (19–73)	48.0 (15–69)	49.0 (15–73)
Men, *n* (%)	58 (65.9)	45 (52.9)	103 (59.5)	51 (66.2)	47 (59.5)	98 (62.8)	31 (66.0)	29 (63.0)	60 (64.5)
Prior aGVHD grade, *n* (%)^a^									
Any	50 (56.8)	49 (57.6)	99 (57.2)	42 (54.5)	39 (49.4)	81 (51.9)	23 (48.9)	22 (47.8)	45 (48.4)
I	9 (10.2)	20 (23.5)	29 (16.8)	16 (20.8)	10 (12.7)	26 (16.7)	11 (23.4)	3 (6.5)	14 (15.1)
II	33 (37.5)	20 (23.5)	53 (30.6)	20 (26.0)	23 (29.1)	43 (27.6)	10 (21.3)	14 (30.4)	24 (25.8)
III	8 (9.1)	7 (8.2)	15 (8.7)	6 (7.8)	5 (6.3)	11 (7.1)	2 (4.3)	4 (8.7)	6 (6.5)
IV	0	2 (2.4)	2 (1.2)	0	1 (1.3)	1 (0.6)	0	1 (2.2)	1 (1.1)
SR-aGVHD	6 (6.8)	8 (9.4)	14 (8.1)	12 (15.6)	9 (11.4)	21 (13.5)	8 (17.0)	8 (17.4)	16 (17.2)
Overall severity of SR-cGVHD at study entry, *n* (%)									
Mild	0	0	0	0	1 (1.3)	1 (0.6)	0	1 (2.2)	1 (1.1)
Moderate	39 (44.3)	36 (42.4)	75 (43.4)	29 (37.7)	37 (46.8)	66 (42.3)	15 (31.9)	20 (43.5)	35 (37.6)
Severe	49 (55.7)	49 (57.6)	98 (56.6)	48 (62.3)	41 (51.9)	89 (57.1)	32 (68.1)	25 (54.3)	57 (61.3)
Time from prior cGVHD diagnosis to randomization, mean (SD), days^b^	196.4 (266.7)	154.3 (182.0)	175.7 (229.4)	274.1 (296.6)	305.8 (353.5)	290.1 (326.0)	300.9 (300.4)	346.0 (349.9)	323.2 (324.8)

*aGVHD* acute graft-versus-host disease, *BAT* best available therapy, *cGVHD* chronic graft-versus-host disease, *SR-aGVHD* steroid-refractory acute graft-versus-host disease, *SR-cGVHD* steroid-refractory chronic graft-versus-host disease, *SD* standard deviation, *y* years.

Race and ethnicity data are reported in REACH3 study [1].

^a^Severity of aGVHD before patients were diagnosed with SR-cGVHD.

^b^Time between diagnosis of initial cGVHD (not steroid refractory) and the randomization for treatment of SR-cGVHD.

Approximately half of patients with cGVHD (*n* = 180/329 [54.7%]) had experienced prior aGVHD. A numerically greater percentage of patients in the early treatment group had prior aGVHD compared with the other 2 groups (57.2%, 51.9%, and 48.4% of patients in the early, late-and-very-late, and very-late subgroups, respectively). At time of study entry, all but 1 patient (in the very-late subgroup) had moderate or severe SR-cGVHD.

### Treatment initiation in patients with SR-aGVHD

**Fig. 1:**
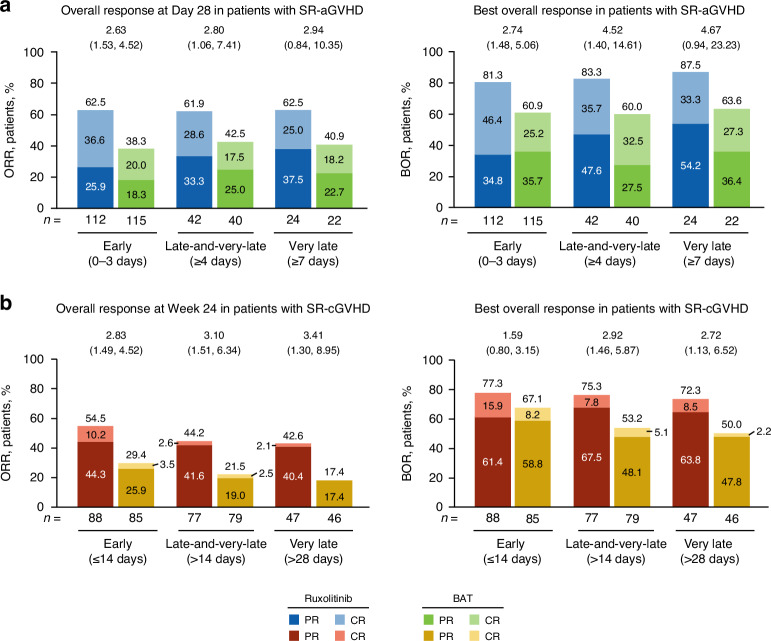
Overall response rate and best overall response by treatment initiation subgroup. **a** ORR: CR + PR at Day 28 (SR-aGVHD). **b** ORR: CR + PR at Week 24 (SR-cGVHD). Values at the top of graphs indicate OR (95% CI) for ruxolitinib vs BAT. BAT best available therapy, CR complete response, OR odds ratio, ORR overall response rate, PR partial response, SR-aGVHD steroid-refractory acute graft-versus-host disease, SR-cGVHD steroid-refractory chronic graft-versus-host disease.

**Fig. 2:**
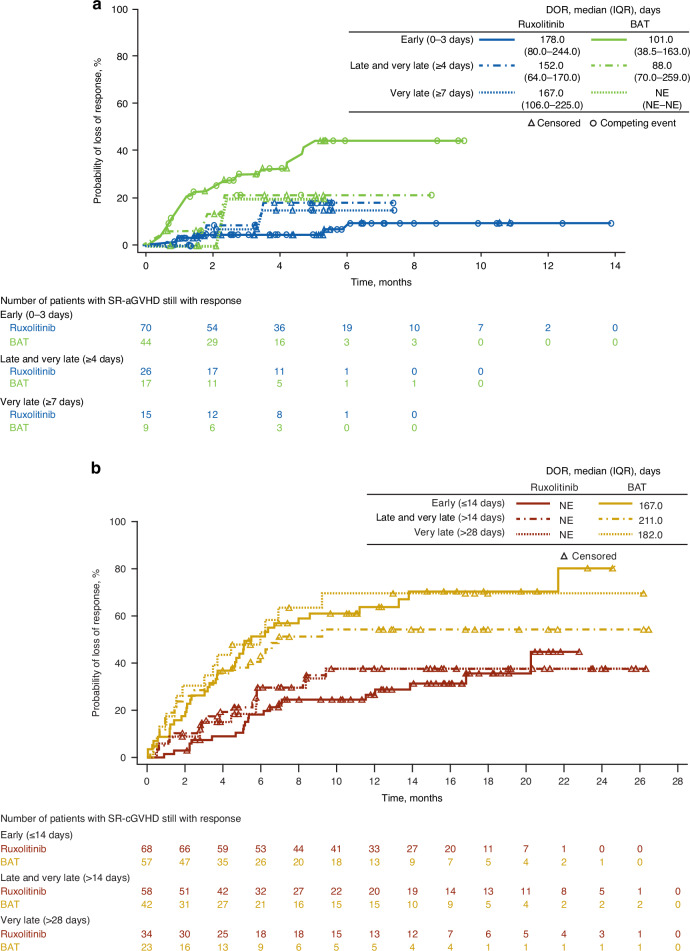
Duration of response in patients treated with ruxolitnib or BAT. **a** DOR in patients with SR-aGVHD. **b** DOR in patients with SR-cGVHD. BAT best available therapy, DOR duration of response, IQR interquartile range, NE not evaluable, SR-aGVHD steroid-refractory acute graft-versus-host disease, SR-cGVHD steroid-refractory chronic graft-versus-host disease.

Among patients with SR-aGVHD, Day 28 ORR was significantly higher with ruxolitinib treatment versus BAT in the early and late-and-very-late subgroups and numerically greater in the very-late treatment subgroup (Fig. 1a). A greater percentage of patients treated with ruxolitinib versus BAT achieved CR in all 3 time-to-treatment subgroups (Fig. 1a). BOR was also significantly higher with ruxolitinib treatment compared with BAT in the early and late-and-very-late subgroups and numerically greater in the very-late treatment subgroup (Fig. 1a). Median DOR was longer with ruxolitinib treatment versus BAT in the early (178.0 vs 101.0 days) and late-and-very-late treatment initiation subgroups (152.0 vs 88.0 days; Fig. 2a).

**Fig. 3:**
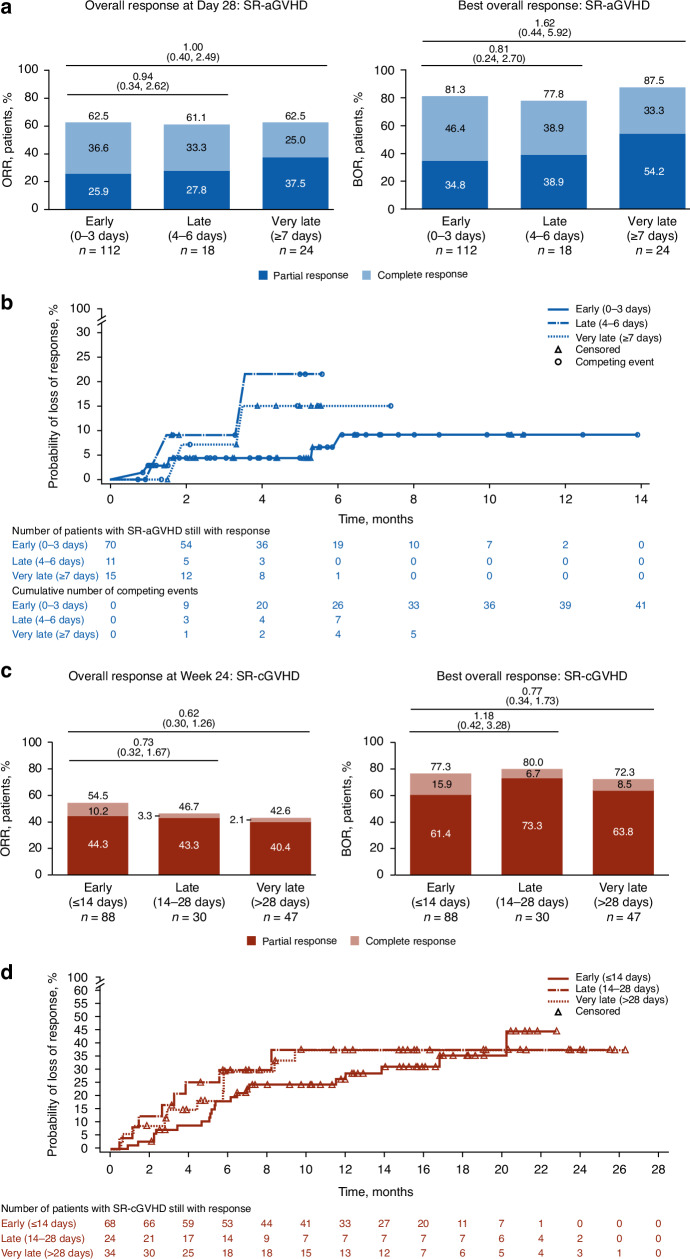
Time to ruxolitinib treatment initiation subgroup comparisons for responses and duration of response. **a** ORR and BOR for patients with SR-aGVHD. **b** DOR for patients with SR-aGVHD. **c** ORR and BOR for patients with SR-cGVHD. **d** DOR for patients with SR-cGVHD. OR odds ratio, SR-aGVHD steroid-refractory acute graft-versus-host disease, SR-cGVHD steroid-refractory chronic graft-versus-host disease. **a**, **c** Values at the top of graphs indicate OR (95% CI) for early versus late or very late treatment initiation.

Responses among early, late, and very-late subgroups were compared in patients with SR-aGVHD treated with ruxolitinib. No significant differences between time-to-treatment subgroups were observed for ORR or BOR, although CR rates were numerically higher in early and late ruxolitinib initiation subgroups versus the very-late subgroup (Fig. 3a). Probability of loss of response was greater with late initiation and very late initiation of ruxolitinib versus early initiation (Fig. 3b).

### Treatment initiation in patients with SR-cGVHD

Among patients with SR-cGVHD, Week 24 ORR was significantly higher with ruxolitinib treatment versus BAT in all 3 time-to-treatment groups (Fig. 1b). BOR was significantly higher in the late-and-very-late and very-late ruxolitinib initiation subgroups with ruxolitinib treatment versus BAT, and numerically higher in the early subgroup (Fig. 1b).

Ruxolitinib treatment increased DOR compared with BAT in patients with SR-cGVHD. Median DOR ranged between 167.0–211.0 days in the BAT subgroups and was not reached in any ruxolitinib subgroup (Fig. 2b).

Responses among the early, late, and very-late subgroups were compared in patients with SR-cGVHD treated with ruxolitinib. No significant differences in ORR, BOR, or DOR were observed among ruxolitinib time-to-treatment subgroups (Fig. 3c, d).

### Cytopenias in patients with SR-aGVHD

**Fig. 4:**
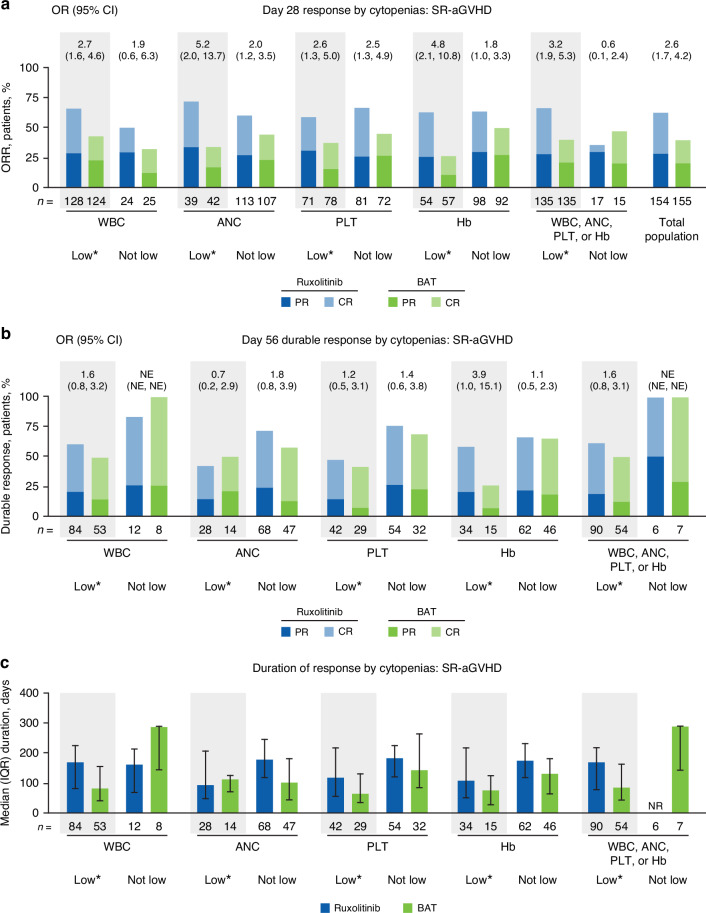
Effect of cytopenias on responses in patients with SR-aGVHD treated with ruxolitinib or BAT. **a** ORR at Day 28. **b** Durable response at Day 56, defined as the proportion of all patients who achieved a CR or PR at Day 28 and maintained a CR or PR at Day 56. **c** Median duration of response. ANC absolute neutrophil count, BAT best available therapy, CR complete response, Hb hemoglobin, IQR interquartile range, NE not evaluable, NR not reached, OR odds ratio, ORR overall response rate, PLT platelet count, PR partial response, SR-aGVHD steroid-refractory acute graft-versus-host disease, WBC white blood cell count. * Cytopenias were defined as low blood counts between baseline and Week 4; low blood cell counts were defined as: WBC, <5 × 10^9^ cells/L; ANC, <1 × 10^9^ cells/L; PLT, <30 × 10^9^ cells/L; Hb, <8 g/dL.

The ORRs for patients with SR-aGVHD were compared among patients stratified by low versus not-low WBC, ANC, PLT, or Hb count. ORRs at Day 28 among patients treated with ruxolitinib were similar between low and not-low WBC, ANC, PLT, or Hb subgroups individually (eg, low PLT, ORR 59.2% [95% CI 46.8–70.7] vs not-low PLT, ORR 66.7% [95% CI 55.3–76.8]). For the period of time that ORR was measured (until Day 28), the most frequent dose reduction was from 20 mg/d to 10 mg/d (44.4% of 135 patients with any cytopenia, vs 17.6% of 17 patients with no cytopenias; Supplementary Fig. 1), although the median dose of ruxolitinib (any cytopenia, 20.0 mg/d; no cytopenia, 20.0 mg/d; Q1–Q3 of median doses across all 152 patients with or without cytopenias, 17.85–20.0 mg/d) was approximately equal to the 20 mg/d starting dose in all subgroups regardless of cytopenia status. The presence of any cytopenia (low WBC, ANC, PLT, or Hb) did not diminish favorable response rates among patients treated with ruxolitinib (cytopenia subgroup, ORR 66.7% [95% CI 58.0–74.5] vs no cytopenia subgroup, ORR 35.3% [95% CI 14.2–61.7]; Fig. 4a). Response rate was numerically, but not significantly, higher for BAT than ruxolitinib in patients with no cytopenias (*n* = 6/17 [35.3%] treated with ruxolitinib; *n* = 7/15 [46.7%] treated with BAT). However, response rates were numerically or significantly higher in patients treated with ruxolitinib versus BAT for all cytopenia subgroups and for patients overall (Fig. 4a).

**Fig. 5:**
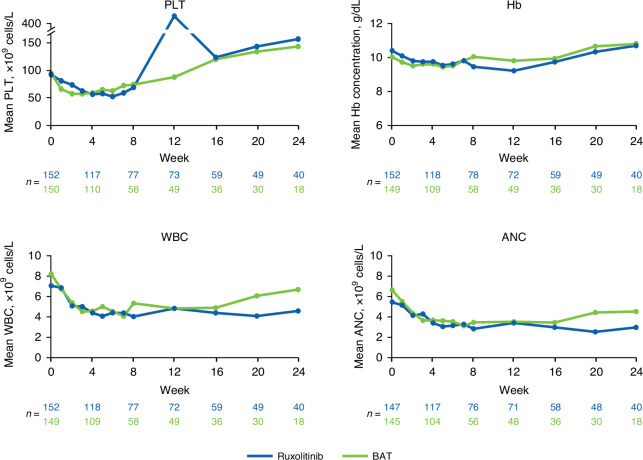
Hematologic laboratory values in patients with SR-aGVHD. ANC absolute neutrophil count, BAT best available therapy, Hb hemoglobin, PLT platelet count, SR-aGVHD steroid-refractory acute graft-versus-host disease, WBC white blood cell count.

Durable responses were observed across all subgroups (Fig. 4b). For the subgroup of patients with any cytopenia, median DOR was numerically longer for patients treated with ruxolitinib versus BAT (Fig. 4c). Mean PLT counts and Hb concentrations decreased over the first 4–12 weeks of treatment, then recovered, and were mostly similar between ruxolitinib and BAT treatment groups through Week 24 (Fig. 5). Mean WBC and ANC counts similarly decreased through Week 8 with both ruxolitinib and BAT and were generally lower in the ruxolitinib versus BAT group through Week 24 (Fig. 5).

## Discussion

### Treatment initiation in SR-aGVHD

Consistent with the overall results of the phase 3 REACH2 trial, these post hoc analyses found that Day 28 ORRs, BORs, CR rates, and DOR were higher for patients treated with ruxolitinib versus BAT [14]. From these analyses, early and late treatment groups had numerically higher CR rates than the very-late treatment group (36.6%, 33.3%, and 25.0%, respectively). These results are consistent with a retrospective analysis that found higher Day 28 CR rates among patients with SR-aGVHD treated with ruxolitinib within 7 days (*n* = 29/42 [69%] ≤7 days [median, 3 days] vs *n* = 5/20 [25%] >7 days, *P* = 0.001) [24]. For patients in REACH2, the lower GI tract was the most commonly affected organ. In a separate study on GI SR-aGVHD, the CR rate was similar to these post hoc analyses (35.1% for 77 patients with GI SR-aGVHD, 57 of whom were treated with ruxolitinib as second-line therapy) [25]. Overall, these findings suggest early treatment with ruxolitinib may provide the most benefit for patients with SR-aGVHD.

### Treatment initiation in SR-cGVHD

Efficacy outcomes (Week 24 ORRs, BORs, and DORs) were also higher in patients treated with ruxolitinib versus those treated with BAT in both the post hoc REACH3 analyses presented here and the phase 3 REACH3 trial [1]. BOR rates were comparable in these post hoc analyses and in a single-center retrospective study of patients with refractory severe cGVHD (*n* = 68/88 [77.3%] for early ruxolitinib treatment initiation versus *n* = 17/23 [74%] treated with ruxolitinib and extracorporeal photopheresis) [26].

In this post hoc analysis of REACH3, timing of ruxolitinib treatment initiation did not affect response rates in patients with SR-cGVHD (Week 24 ORRs and DOR rates were similar among early, late, and very-late treatment initiation groups). However, among patients with SR-cGVHD with a treatment response, median DOR was 5.49–6.93 months for patients treated with BAT and was not reached in any of the ruxolitinib treatment initiation time subgroups, suggesting a more durable response with ruxolitinib regardless of treatment initiation time.

### Cytopenias in patients with SR-aGVHD

Cytopenias are common adverse events in patients with GVHD, regardless of treatment, and are associated with worse outcomes [1, 14, 27–29]. Certain circumstances introduce additional confounding variables; for example, ganciclovir treatment for cytomegalovirus reactivation has been associated with neutropenia and thrombocytopenia [30]. Additionally, GVHD pathogenesis may also contribute to the frequency of cytopenias: aGVHD/cGVHD are independent risk factors for development of thrombocytopenia after HSCT [31]. Thus, understanding the impact of cytopenias on efficacy for both ruxolitinib and BAT is important.

The post hoc analysis of REACH2 indicated that the presence of low ANC, WBC, PLT counts, or Hb concentrations did not affect the favorable efficacy outcomes (ORR/DOR) in the treatment of SR-aGVHD. Patients were able to maintain the starting dose of ruxolitinib during the period in which Day 28 response rates were measured. Occurrence of dose reductions had minimal impact on patients’ response to ruxolitinib regardless of cytopenia status. Ruxolitinib was associated with statistically higher Day 28 response rates than BAT for most low and not-low blood count groups and for the full patient population overall, with durable responses observed at Day 56.

Ruxolitinib and BAT were associated with similar reductions in blood counts early in treatment. These findings highlight the potential myelosuppressive effects of aGVHD development related to the hyperinflammatory state and increased production of cytokines versus the impact of treatment on blood counts. These results are consistent with those reported in exploratory analyses of pooled data from the COMFORT studies, in which ruxolitinib-associated anemia, which generally occurs during early therapy, did not predict shortened survival [32, 33].

### Study limitations

Limitations of this study include low patient numbers in each of the non-prespecified subgroups, the lack of blinding in the REACH2 and REACH3 studies, and the hypothesis-generating nature of the post hoc analyses.

## Conclusions

These post hoc analyses of the REACH2 and REACH3 trials suggest that ruxolitinib may provide more robust clinical benefit compared with BAT for patients with SR-aGVHD or SR-cGVHD, regardless of treatment initiation time after diagnosis of SR disease. In patients with SR-aGVHD, response rates were higher with early ruxolitinib treatment, reinforcing the importance of initiation of early treatment in SR-aGVHD. However, studies with a larger patient population are needed to confirm this observation. In addition, the presence of cytopenias did not affect the beneficial efficacy outcomes of ruxolitinib versus BAT in patients with SR-aGVHD. Furthermore, these patients were able to maintain dose intensity of ruxolitinib throughout the 28-day period of the primary endpoint. Timing of ruxolitinib treatment initiation did not affect response rates or DOR in patients with SR-cGVHD.

These findings suggest that physicians treating HSCT recipients should consider initiating ruxolitinib treatment within 3 days of SR-aGVHD diagnosis to optimize response rates and durability of response. Maintaining dose intensity during ruxolitinib treatment is feasible, appears to provide better efficacy versus BAT in SR-aGVHD or SR-cGVHD, and is manageable with appropriate supportive care to achieve desired treatment efficacy outcomes.

## Supplementary information

Supplemental Material

## Data Availability

Incyte (Wilmington, DE, USA) is committed to data sharing that advances science and medicine while protecting patient privacy. Qualified external scientific researchers may request anonymized datasets owned by Incyte for the purpose of conducting legitimate scientific research. Researchers may request anonymized datasets from any interventional study (except Phase 1 studies) for which the product and indication have been approved on or after 1 January 2020 in at least one major market (e.g. US, EU, JPN). Data will be available for request after the primary publication or 2 years after the study has ended. Information on Incyte’s clinical trial data sharing policy and instructions for submitting clinical trial data requests are available at: https://www.incyte.com/Portals/0/Assets/Compliance%20and%20Transparency/clinical-trial-data-sharing.pdf?ver=2020-05-21-132838-960.

## References

[1] Zeiser R, Polverelli N, Ram R, Hashmi SK, Chakraverty R, Middeke JM, et al. Ruxolitinib for glucocorticoid-refractory chronic graft-versus-host disease. N Engl J Med. 2021;385:228–38. 34260836 10.1056/NEJMoa2033122

[2] Zeiser R, Blazar BR. Acute graft-versus-host disease - biologic process, prevention, and therapy. N Engl J Med. 2017;377:2167–79. 29171820 10.1056/NEJMra1609337 PMC6034180

[3] McDonald GB, Sandmaier BM, Mielcarek M, Sorror M, Pergam SA, Cheng GS, et al. Survival, nonrelapse mortality, and relapse-related mortality after allogeneic hematopoietic cell transplantation: comparing 2003-2007 versus 2013-2017 cohorts. Ann Intern Med. 2020;172:229–39. 31958813 10.7326/M19-2936 PMC7847247

[4] Jagasia M, Zeiser R, Arbushites M, Delaite P, Gadbaw B, Bubnoff NV. Ruxolitinib for the treatment of patients with steroid-refractory GVHD: an introduction to the REACH trials. Immunotherapy. 2018;10:391–402. 29316837 10.2217/imt-2017-0156

[5] Zeiser R, Teshima T. Nonclassical manifestations of acute GVHD. Blood. 2021;138:2165–72. 34482399 10.1182/blood.2021012431

[6] Center for International Blood & Marrow Transplant Research (CIBMTR). HCT trends and survival data. Available at: https://www.cibmtr.org/referencecenter/slidesreports/summaryslides/Pages/index.aspx. Accessed August 2, 2023.

[7] Dignan FL, Clark A, Amrolia P, Cornish J, Jackson G, Mahendra P, et al. Diagnosis and management of acute graft-versus-host disease. Br J Haematol. 2012;158:30–45. 22533831 10.1111/j.1365-2141.2012.09129.x

[8] Martin PJ, Rizzo JD, Wingard JR, Ballen K, Curtin PT, Cutler C, et al. First- and second-line systemic treatment of acute graft-versus-host disease: recommendations of the American Society of Blood and Marrow Transplantation. Biol Blood Marrow Transplant. 2012;18:1150–63. 22510384 10.1016/j.bbmt.2012.04.005 PMC3404151

[9] Ruutu T, Gratwohl A, de Witte T, Afanasyev B, Apperley J, Bacigalupo A, et al. Prophylaxis and treatment of GVHD: EBMT-ELN working group recommendations for a standardized practice. Bone Marrow Transplant. 2014;49:168–73. 23892326 10.1038/bmt.2013.107

[10] MacMillan ML, Weisdorf DJ, Wagner JE, DeFor TE, Burns LJ, Ramsay NK, et al. Response of 443 patients to steroids as primary therapy for acute graft-versus-host disease: comparison of grading systems. Biol Blood Marrow Transplant. 2002;8:387–94. 12171485 10.1053/bbmt.2002.v8.pm12171485

[11] Westin JR, Saliba RM, De Lima M, Alousi A, Hosing C, Qazilbash MH, et al. Steroid-refractory acute GVHD: predictors and outcomes. Adv Hematol. 2011;2011:601953. 22110505 10.1155/2011/601953 PMC3216266

[12] Olivieri A, Cimminiello M, Corradini P, Mordini N, Fedele R, Selleri C, et al. Long-term outcome and prospective validation of NIH response criteria in 39 patients receiving imatinib for steroid-refractory chronic GVHD. Blood. 2013;122:4111–8. 24152907 10.1182/blood-2013-05-494278

[13] Weng JY, Du X, Geng SX, Peng YW, Wang Z, Lu ZS, et al. Mesenchymal stem cell as salvage treatment for refractory chronic GVHD. Bone Marrow Transplant. 2010;45:1732–40. 20818445 10.1038/bmt.2010.195 PMC3035976

[14] Zeiser R, von Bubnoff N, Butler J, Mohty M, Niederwieser D, Or R, et al. Ruxolitinib for glucocorticoid-refractory acute graft-versus-host disease. N Engl J Med. 2020;382:1800–10. 32320566 10.1056/NEJMoa1917635

[15] Martini DJ, Chen YB, DeFilipp Z. Recent FDA approvals in the treatment of graft-versus-host disease. Oncologist. 2022;27:685–93. 35443042 10.1093/oncolo/oyac076 PMC9355804

[16] National Comprehensive Cancer Network. NCCN clinical practice guidelines in oncology. *Hematopoietic cell transplantation (HCT) (Version 1.2023)*. Available at: https://www.nccn.org/guidelines/guidelines-detail?category=3&id=1501. Accessed July 19, 2023.

[17] Palmer J, Chai X, Pidala J, Inamoto Y, Martin PJ, Storer B, et al. Predictors of survival, nonrelapse mortality, and failure-free survival in patients treated for chronic graft-versus-host disease. Blood. 2016;127:160–6. 26527676 10.1182/blood-2015-08-662874 PMC4705606

[18] Fan S, Huo W-X, Yang Y, Shen M-Z, Mo X-D. Efficacy and safety of ruxolitinib in steroid-refractory graft-versus-host disease: a meta-analysis. Front Immunol. 2022;13:954268. 35990629 10.3389/fimmu.2022.954268 PMC9386528

[19] Zeiser R, Russo D, Ram R, Hashmi S, Ronjon C, Middeke JM et al. Ruxolitinib in Patients With Chronic Graft-Versus-Host-Disease: 3-Year Final Analysis of Efficacy and Safety From the Phase III REACH3 Study. *In: 65th ASH Annual Meeting and Exposition*; 2023 December 9-12; San Diego, CA; 2023.

[20] Koreth J, Kim HT, Jones KT, Lange PB, Reynolds CG, Chammas MJ, et al. Efficacy, durability, and response predictors of low-dose interleukin-2 therapy for chronic graft-versus-host disease. Blood. 2016;128:130–7. 27073224 10.1182/blood-2016-02-702852 PMC4937358

[21] Deeg HJ. How I treat refractory acute GVHD. Blood. 2007;109:4119–26. 17234737 10.1182/blood-2006-12-041889 PMC1885485

[22] Martin PJ, Lee SJ, Przepiorka D, Horowitz MM, Koreth J, Vogelsang GB, et al. National Institutes of Health consensus development project on criteria for clinical trials in chronic graft-versus-host disease: VI. The 2014 clinical trial design working group report. Biol Blood Marrow Transplant. 2015;21:1343–59. 25985921 10.1016/j.bbmt.2015.05.004 PMC4506719

[23] Müskens KF, Lindemans CA, Dandis R, Nierkens S, Belderbos ME. Definitions, incidence and outcome of poor graft function after hematopoietic cell transplantation: a systematic review and meta-analysis. Blood Rev. 2023;60:101076. 36990959 10.1016/j.blre.2023.101076

[24] Ren J, Lin K, Xu J, Lu Q, Luo Y, Lin C, et al. When is the best time and grade to start ruxolitinib in corticosteroid‐refractory acute graft‐versus‐host‐disease: A multi‐center research. Clin Transplant 2023: e15195. 10.1111/ctr.15195 37987525

[25] Biavasco F, Ihorst G, Wäsch R, Wehr C, Bertz H, Finke J, et al. Therapy response of glucocorticoid-refractory acute GVHD of the lower intestinal tract. Bone Marrow Transplant. 2022;57:1500–6. 35768570 10.1038/s41409-022-01741-3 PMC9532244

[26] Maas-Bauer K, Kiote-Schmidt C, Bertz H, Apostolova P, Wäsch R, Ihorst G, et al. Ruxolitinib–ECP combination treatment for refractory severe chronic graft-versus-host disease. Bone Marrow Transplant. 2021;56:909–16. 33203951 10.1038/s41409-020-01122-8

[27] Arora M, Klein JP, Weisdorf DJ, Hassebroek A, Flowers ME, Cutler CS, et al. Chronic GVHD risk score: a Center for International Blood and Marrow Transplant research analysis. Blood. 2011;117:6714–20. 21493797 10.1182/blood-2010-12-323824 PMC3123030

[28] Kim DH, Sohn SK, Jeon SB, Baek JH, Kim JG, Lee NY, et al. Prognostic significance of platelet recovery pattern after allogeneic HLA-identical sibling transplantation and its association with severe acute GVHD. Bone Marrow Transplant. 2006;37:101–8. 16258533 10.1038/sj.bmt.1705203

[29] Zeiser R, Socié G, Schroeder MA, Abhyankar S, Vaz CP, Kwon M, et al. Efficacy and safety of itacitinib versus placebo in combination with corticosteroids for initial treatment of acute graft-versus-host disease (GRAVITAS-301): a randomised, multicentre, double-blind, phase 3 trial. Lancet Haematol. 2022;9:e14–e25. 34971577 10.1016/S2352-3026(21)00367-7

[30] Winston DJ, Ho WG, Bartoni K, Du Mond C, Ebeling DF, Buhles WC, et al. Ganciclovir prophylaxis of cytomegalovirus infection and disease in allogeneic bone marrow transplant recipients: results of a placebo-controlled, double-blind trial. Ann Intern Med. 1993;118:179–84. 8380243 10.7326/0003-4819-118-3-199302010-00004

[31] Wang H, Qi J, Li X, Chu T, Qiu H, Fu C, et al. Prognostic value of thrombocytopenia in myelodysplastic syndromes after hematopoietic stem cell transplantation. Front Oncol. 2022;12:940320. 35898899 10.3389/fonc.2022.940320 PMC9309887

[32] Gupta V, Harrison C, Hexner EO, Al-Ali HK, Foltz L, Montgomery M, et al. The impact of anemia on overall survival in patients with myelofibrosis treated with ruxolitinib in the COMFORT studies. Haematologica. 2016;101:e482–e484. 27587385 10.3324/haematol.2016.151449 PMC5479619

[33] Palandri F, Breccia M, Mazzoni C, Auteri G, Elli EM, Trawinska MM, et al. Ruxolitinib in cytopenic myelofibrosis: response, toxicity, drug discontinuation, and outcome. Cancer. 2023;129:1704–13. 36932983 10.1002/cncr.34722

