# Supplementary material for: Impact of cytopenias and early versus late treatment with ruxolitinib in patients with steroid-refractory acute or chronic graft-versus-host disease
Source: Bone Marrow Transplant. 2024 Nov 6;60(1):69–78. doi: 10.1038/s41409-024-02445-6 (PMC11726446; doi:10.1038/s41409-024-02445-6)
Supplement: Supplementary file 1 — Supplemental Material [file 41409_2024_2445_MOESM1_ESM.docx]

**SUPPLEMENTARY MATERIAL**

**Impact of Cytopenias and Early Versus Late Treatment With Ruxolitinib in Patients With Steroid-Refractory Acute or Chronic Graft-Versus-Host Disease**

Zahra Mahmoudjafari, PharmD,^1^ Valkal Bhatt, PharmD,^2^ John Galvin, MD, MS, MPH,^2^ Zhenyi Xue, PhD,^2^ Robert Zeiser, MD,^3^ Franco Locatelli, MD,^4,5^ Gérard Socié, MD, PhD,^6^ Mohamad Mohty, MD^7^

^1^University of Kansas Cancer Center, Westwood, KS, USA; ^2^Incyte Corporation, Wilmington, DE, USA; ^3^University Medical Center Freiburg, Freiburg, Germany; ^4^IRCCS Ospedale Pediatrico Bambino Gesù, Rome, Italy; ^5^Catholic University of the Sacred Heart, Rome, Italy; ^6^Hôpital Saint-Louis Hematology Transplantation & University Paris Cité, Paris, France; ^7^Hôpital Saint-Antoine Hospital and Sorbonne University, Paris, France

**Figure S1. Ruxolitinib Dose Levels Received at any Time up to Day 28**

**
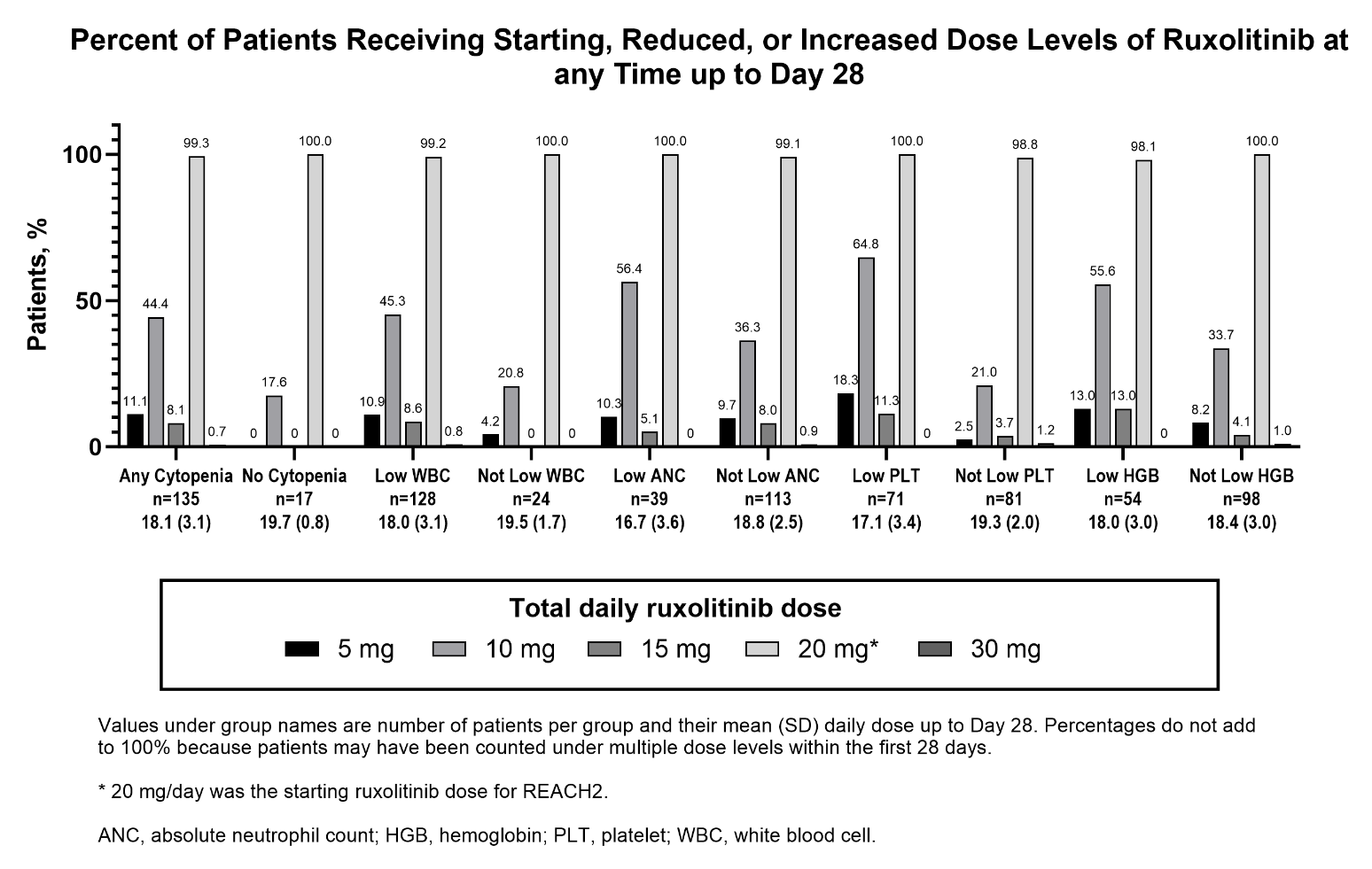
**
